# Pronounced and reversible modulation of the piezoelectric coefficients by a low magnetic field in a magnetoelectric PZT-5%Fe_3_O_4_ system

**DOI:** 10.1038/s41598-019-38675-8

**Published:** 2019-02-18

**Authors:** G. Vertsioti, S. J. Zhang, D. Stamopoulos

**Affiliations:** 1 Department of Solid State Physics, National and Kapodistrian University of Athens, Zografou Panepistimioupolis, Athens, Greece; 2 Institute of Nanoscience and Nanotechnology, National Center for Scientific Research ‘Demokritos’, Aghia Paraskevi, Athens, Greece; 3 Institute for Superconducting and Electronic Materials, Australian Institute of Innovative Materials, University of Wollongong, Wollongong, Australia

## Abstract

Composite magnetoelectric compounds that combine ferroelectricity/piezoelectricity and ferromagnetism/magnetostriction are investigated intensively for room-temperature applications. Here, we studied bulk composites of a magnetostrictive constituent, ferromagnetic Fe_3_O_4_ nanoparticles, homogeneously embedded in a ferroelectric/piezoelectric matrix, Pb(Zr_0.52_Ti_0.48_)O_3_ (PZT). Specifically, we focused on PZT-5%Fe_3_O_4_ samples which are strongly insulating and thus sustain a relatively high out-of-plane external electric field, E_ex,z_. The in-plane strain-electric field curve (S(E_ex,z_)) was carefully recorded upon successive application and removal of an out-of-plane external magnetic field, H_ex,z_. The obtained S(E_ex,z_) data exhibited two main features. First, the respective in-plane piezoelectric coefficients, d(E_ex,z_) = 200–250 pm/V, show a dramatic decrease, 50–60%, upon application of a relatively low H_ex,z_ = 1 kOe. Second, the process is completely reversible since the initial value of d(E_ex,z_) is recovered upon removal of H_ex,z_. Polarization data, P(E_ex,z_), evidenced that the Fe_3_O_4_ nanoparticles introduced *static structural disorder* that made PZT harder. Taken together, these results prove that the Fe_3_O_4_ nanoparticles, except for *static structural disorder*, introduce *reconfigurable magnetic disorder* that modifies the in-plane S(E_ex,z_) curve and the accompanying d(E_ex,z_) of PZT when an external magnetic field is applied at will. The room-temperature feasibility of these findings renders the PZT-x%Fe_3_O_4_ system a solid basis for the development of magnetic-field-controlled PE devices.

## Introduction

Electric field controlled phenomena have been extensively investigated, both experimentally and theoretically, during the past two decades in pristine compounds and composite systems that exhibit complex behavior in many of their physical properties (particle dynamics/interactions, optical, magnetic etc.) motivated by either extrinsic or intrinsic mechanism^1–5^. Regarding particle dynamics/interactions and the accompanying phase transitions, K. Kang studied in detail^1^ systems of highly charged rod-like colloids. They showed that the particle interactions can be controlled by the application of an electric field, thus the phase diagram of relevant systems can be modulated at will. Referring to the optical properties, in a very recent work^2^, S. Shian *et al*. described the correlation between electric field induced surface instabilities and light scattering for a compliant mat of carbon nanotubes on a smooth elastometer bilayer attached to an InSnO coated glass substrate. Interestingly, they observed that increasing the applied electric field, the optical transmittance decreases substantially, in a reversible way, a fact that can give rise to a wide spectrum of applications. Concerning the magnetic properties L. Bai *et al*. recently investigated^3^ boron nitride nanotubes and proved that the application of an electric field causes reconstruction of the electronic bands, ultimately enabling us to transit between phases of different transport and magnetic properties. A relevant wide class of materials, either single phase or composites, are the so-called magnetoelectric (ME). In these materials the electric and magnetic polarization are coupled so that the application of an electric/magnetic field can control their magnetic/electric properties. Notably, the composite ME materials that are constructed of distinct ferroelectric (FE) and ferromagnetic (FM) building units can be designed at will and exhibit better performance when compared to the respective single phase ones. Specifically, ME materials that combine piezoelectric (PE) and FM constituents have recently earned great interest, since the coupling of their properties leads to large ME response upon the appearance of strain via an electric field, even at room temperature, a fact that is rarely met in single phase candidates^4,5^. For this reason, they find tremendous applications in functional devices such as information storage and magnetic field sensors^6–14^. Among this wide class of materials, the PE compound family Pb(Zr_x_Ti_1−x_)O_3_ has been widely investigated, with special interest in the stoichiometry x ≈ 0.52, i.e. the Pb(Zr_0.52_Ti_0.48_)O_3_ compound, that is the so-called morphotropic phase boundary^15–21^. Due to the coexistence of different crystallographic phases the specific Pb(Zr_0.52_Ti_0.48_)O_3_ compound exhibits the maximum possible PE coefficients, d_ij_. As a result, Pb(Zr_x_Ti_1−x_)O_3_, in general, and Pb(Zr_0.52_Ti_0.48_)O_3_, in particular, is widely employed as the PE constituent in ME composites, together with ferro/ferri-magnetic alloys and oxides, like CoFe_2_O_4_, Ni_0.93_Co_0.02_Mn_0.05_Fe_1.95_O_4_, Ni_50_Mn_35_In_15_, Co_1−x_Zn_x_Fe_2_O_4_ (x = 0.0–0.6), Ni_1−x_Zn_x_Fe_2_O_4_ (x = 0.0–0.5), CoFeO_4_^22–27^.

The above mentioned studies^22–27^, as well as the majority of studies concerning ME composites^28–32^, examine the electric-field control of the magnetic properties of the composites. The inverse, i.e. the control of FE or PE properties by an external magnetic field, H_ex_, has been less investigated^33–36^, focusing mostly on the electric polarization. Corresponding studies, performed by means of piezoresponse force microscopy, demonstrate the modulation of FE domains, upon application of an H_ex_ on the order of 2–9 kOe. Among them, Evans *et al*.^35^ and Xie *et al*.^36^ based on Pb(Zr_1−x_,Ti_x_)O_3_ as PE component, studied the changes in polarization^35^ and piezoresponse^36^ upon H_ex_, for the cases of the ME composites PbZr_0.53_Ti_0.47_O_3_-PbFe_0.5_Ta_0.5_O_3_ (PZTFT) single crystals and PbZrTiO-TbDyFe (PZT-TDF) bilayer, respectively. These changes, were significant in magnitude, although partially reversible^35^ or irreversible at all^36^.

Apart from the polarization and piezoresponse, another basic PE parameter, the PE coefficients d_ij_, have not been investigated upon application of an H_ex_, until now. Here, we study the bulk composite system consisting of PE Pb(Zr_0.52_Ti_0.48_)O_3_ (with composition at the so-called morphotropic phase boundary (MPB); called simply PZT for the rest of the paper) and FM Fe_3_O_4_ nanoparticles (NPs) that have noticeable magnetostrictive nature. Specifically, the FM NPs are embedded in the PE matrix in the desired weight percentage PZT-5%Fe_3_O_4_. We focus on the variation of the in-plane PE coefficients upon application of an E_ex,z_ (ranging within ± 10 kV/cm) and a relatively low H_ex,z_ (1 kOe), that are both applied out-of-plane at room temperature. By means of a method based on optical microscopy^37,38^, we showed in a direct way that the application of such a low H_ex_ = 1 kOe, causes a pronounced decrease of the in-plane PE coefficients on the order of 50–60%, that intriguingly is restored upon successive removal of H_ex_. Polarization data showed that with the addition of the Fe_3_O_4_ NPs, PZT becomes harder when compared to its plain form. This is a proof that the FM NPs act as structural disorder that pins the FE domain walls efficiently, a fact that can be attributed to the similar size (50–100 nm) of the FM Fe_3_O_4_ NPs (see ‘Sample preparation’ in ‘METHODS’) and the FE domains of PZT (see^17,39–41^). These combined results prove that the FM Fe_3_O_4_ NPs serve not only as a simple *static structural disorder* but, most important, as a *reconfigurable magnetic disorder* that can be applied at will by means of an external magnetic field to control the PE properties of PZT. Based on the strong PE character of PZT and the noticeable magnetostrictive nature of Fe_3_O_4_, these findings can be ascribed to a strain transfer mechanism realized at the interface of the FM Fe_3_O_4_ NPs and the surrounding FE PZT matrix. Consequently, the quantitatively significant and qualitatively reversible modulation of the in-plane PE coefficients render PZT-5%Fe_3_O_4_ a candidate ME material for applications in functional devices with high ME performance.

## Results and Discussion

**Figure 1:**
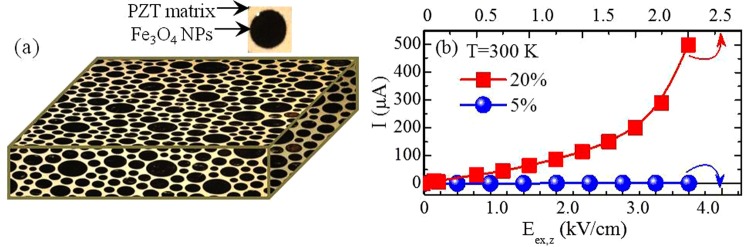
Schematic illustration and electric properties of the PZT-x%Fe_3_O_4_ samples. (**a**) Schematic illustration of the bulk PZT-x%Fe_3_O_4_ samples indicating that the Fe_3_O_4_ FM NPs are embedded in the PZT PE matrix. (**b**) Electric current leakage, I, under the application of an out-of-plane electric field, E_ex,z_, for two PZT-xFe_3_O_4_ samples, with x = 5% and 20%. The bottom/top horizontal axis corresponds to the data referring to x = 5%/x = 20% sample.

A complete series of PZT-xFe_3_O_4_ samples (x = 0–50% w∕w), that is Fe_3_O_4_ NPs homogeneously embedded in the PZT matrix as schematically shown in Fig. 1(a), was preliminary investigated to choose the appropriate insulating sample for the subsequent study. Obviously, a basic criterion is that the sample should remain strongly insulating for a reasonable range of electric field values. Figure 1(b), presents the leakage current as function of an electric field applied out-of-plane, E_ex,z_, for two specific samples with x = 5% and x = 20%. We clearly see that the sample with x = 5% remains insulating, while the sample with x = 20% exhibits a noticeable leakage current for a comparatively much lower value of E_ex,z_. Accordingly, all the experimental results presented below refer to PZT-5%Fe_3_O_4_.

**Figure 2:**
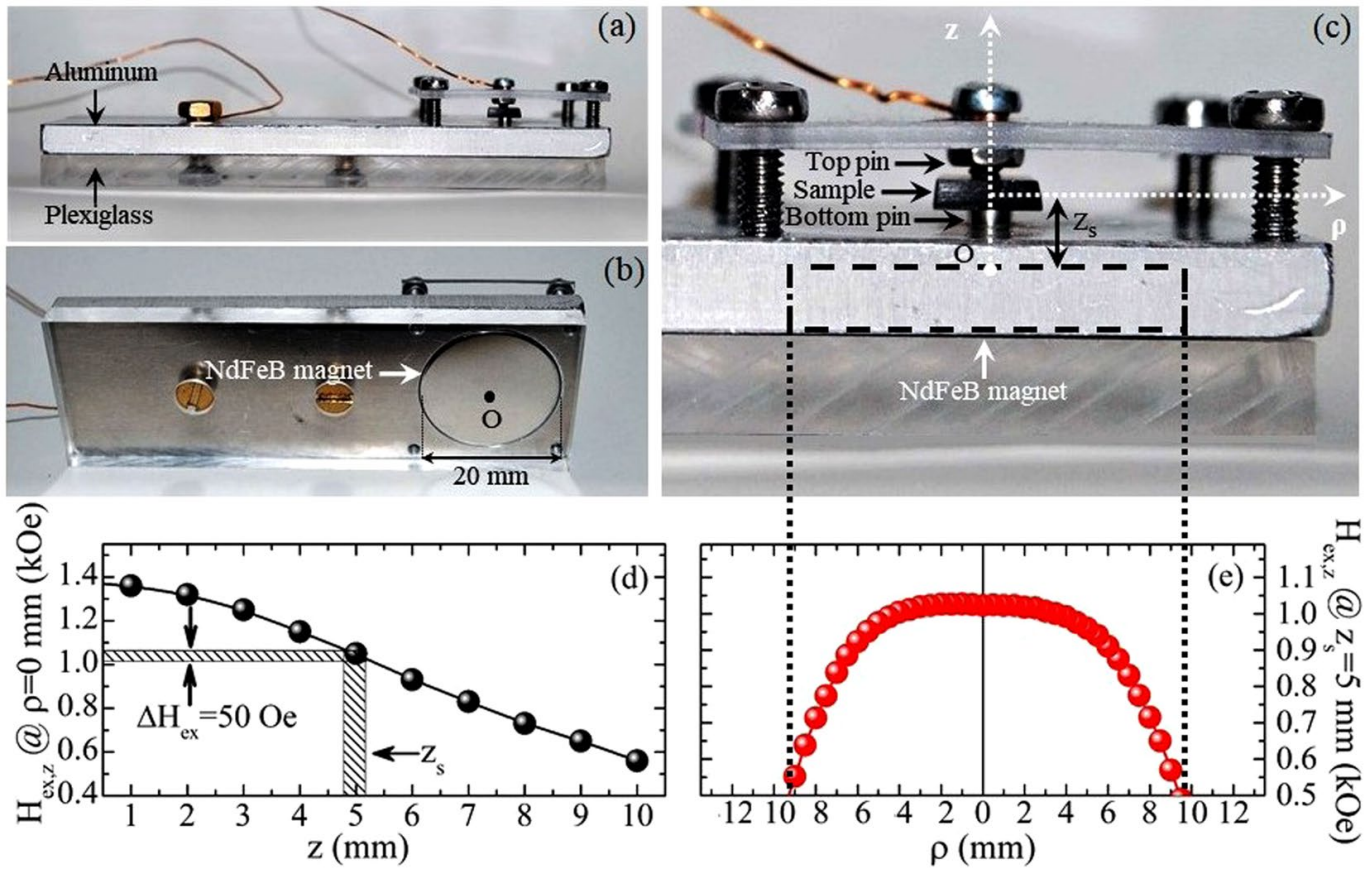
Home-made platform for the simultaneous application of the out-of-plane electric and magnetic fields, E_ex,z_ and H_ex,z_. (**a**) Side view and (**b**) perspective bottom view of the home-made platform constructed for the *local* observation of the sample, by means of an OM, under simultaneous application of an electric field, E_ex,z_, produced by a DC-voltage supply and a constant magnetic field, H_ex,z_, produced by a disc-shape NdFeB permanent magnet. E_ex,z_ can be varied continuously within −10 kV/cm to +10 kV/cm during a set of measurements, while H_ex,z_ is constant, 1 kOe, and exhibits azimuthal symmetry. (**c**) Detail of (**a**) in the sample area. The top and bottom pins hold the sample at its center so that the entire sample is left free to deform. The black dashed rectangle denotes the NdFeB magnet that is embedded in the aluminum platform, with its center, O, aligned with that of the sample (vertical dotted lines denote the boundary of the permanent magnet). The coordinate axes z and ρ are shown with white dotted lines. (**d**) Variation of H_ex,z_ along the z coordinate (ρ = 0 mm). (**e**) Variation of H_ex,z_ along the ρ coordinate (z_s_ = 5 mm).

The constitutive Strain-Electric field curve, S(E_ex,z_), of samples PZT-5%Fe_3_O_4_ was estimated experimentally by using an OM-based technique already discussed in^37,38^ for out-of-plane E_ex,z_ up to 10 kV/cm. Here, we made another modification in the home-made aluminum platform hosting the sample, so that a constant, also out-of-plane magnetic field, H_ex,z_, is applied at will, by a NdFeB permanent magnet, (disc-shaped with diameter 20 mm and thickness 3 mm). The magnet is placed just below the sample as shown in Fig. 2(a–c) and explained in detail at its caption. By means of this experimental setup, the in-plane strain curves, S_zx_(E_ex,z_) and S_zy_(E_ex,z_), can be recorded even under the presence of an H_ex,z_ (see ‘Section I’ in Supplementary Information). We note that all materials used in this construction are non magnetic, else the magnetic field produced by the NdFeB permanent magnet would be non-homogeneous. Since our study aims to investigate the PE response upon the absence/presence of an external magnetic field, the homogeneity of H_ex,z_ is crucial. In this context, accurate mapping of H_ex,z_ was performed along the z coordinate, as shown in Fig. 2(d), and along the ρ coordinate, as shown in Fig. 2(e) (vertical dotted lines denote the boundary of the permanent magnet). The sample is placed at z_s_ = 5 mm, with its center aligned with that of the magnet. Accordingly, the data of Fig. 2(d,e) prove that the external magnetic field of value H_ex,z_(z_s_) = 1 kOe is fairly homogeneous over the entire volume of the PZT-5%Fe_3_O_4_ sample. This magnetic field brings the FM Fe_3_O_4_ NPs close to saturation (see magnetization data, below).

**Figure 3:**
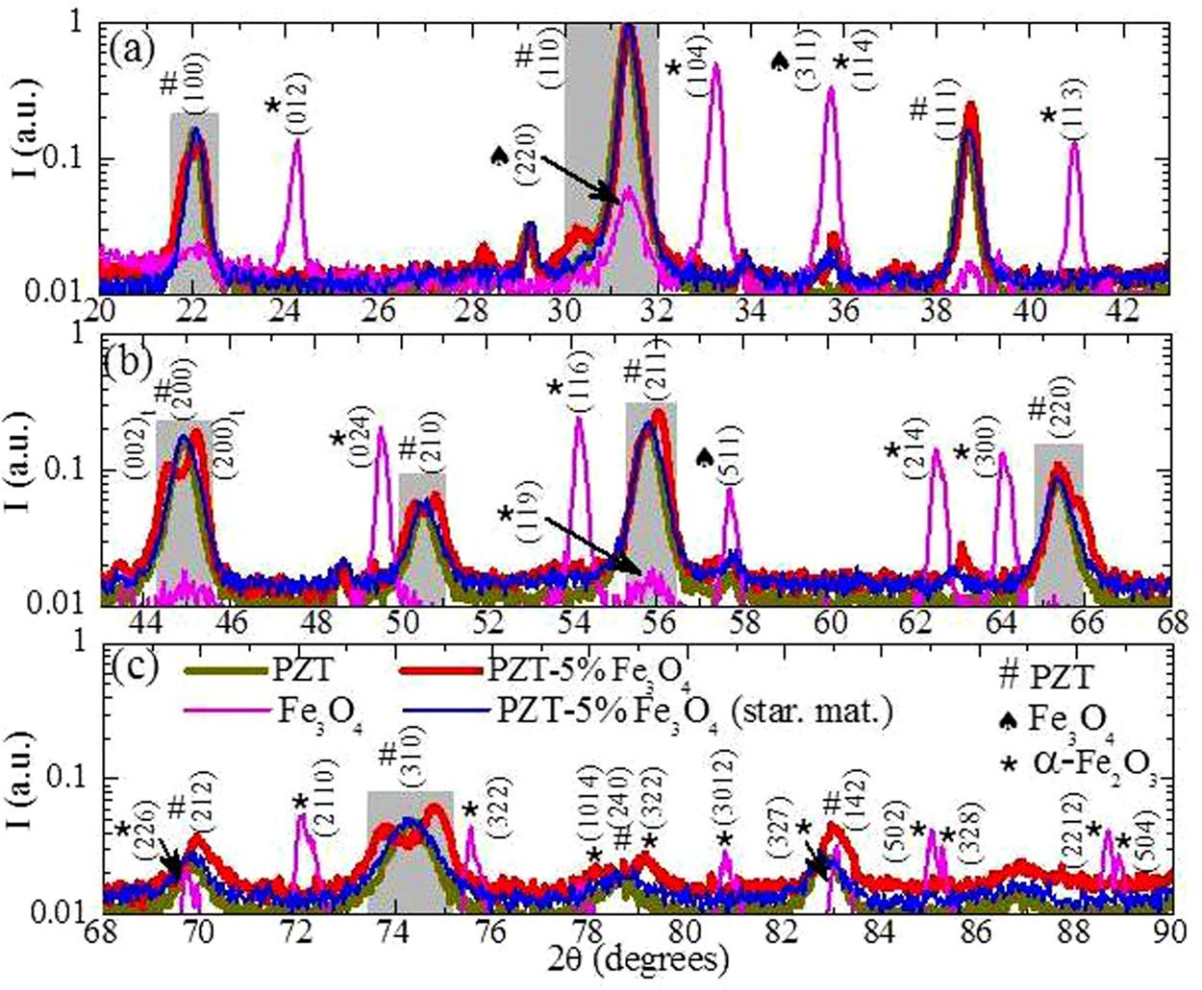
Characterization of crystallographic structure for non-sintered PZT-5%Fe_3_O_4_, sintered PZT, sintered Fe_3_O_4_ NPs, and sintered PZT-5%Fe_3_O_4_ samples. (**a**–**c**) XRD spectra presented in the 2θ range 20–90° for non-sintered PZT-5%Fe_3_O_4_ (starting material: star. mat.), sintered PZT, sintered Fe_3_O_4_ NPs, and the sintered PZT-5%Fe_3_O_4_ composite. The assignment of newly emerging peaks evidences the formation of hematite, α-Fe_2_O_3_, by the oxidation of the magnetite, Fe_3_O_4_, NPs. The assignment of broadened/splitted peaks (highlighted by the grey-shaded areas at 2θ = 22°, 31°, 45°, 50.5°, 56°, 65.5° and 74°) proves that PZT hosts both the rhombohedral and the tetragonal phases.

Detailed XRD data is shown in Fig. 3(a–c) on a comparative basis, with assignment of the main peaks, for non-sintered PZT-5%Fe_3_O_4_ (starting material: star. mat.), sintered PZT, sintered Fe_3_O_4_ NPs, and the sintered PZT-5%Fe_3_O_4_ composite (sintering conditions: T = 1000 °C, for t = 2 h in air). These results prove that (i) the magnetite, Fe_3_O_4_, is oxidized to hematite, α-Fe_2_O_3_, (partially, as evidenced by the magnetization data of Fig. 5, below), (ii) the MPB compositional PZT (Pb(Zr_0.52_Ti_48_)O_3_) undergoes a phase transition, probably promoted by the presence of Fe_3_O_4_, resulting in a coexistence of rhombohedral and tetragonal phases (evidenced by the peak broadening/splitting observed in the vicinity of 2θ = 22°, 31°, 45°, 50.5°, 56°, 65.5° and 74°, as highlighted by the grey-shaded areas)^20,42,43^ and (iii) no byproducts/new compounds are present so that the former PZT-5%Fe_3_O_4_ system evolves to a PZT-5%(Fe_3_O_4_/α-Fe_2_O_3_) composite (for clarity, simply denoted PZT-5%Fe_3_O_4_ in the rest of the paper).

**Figure 4:**
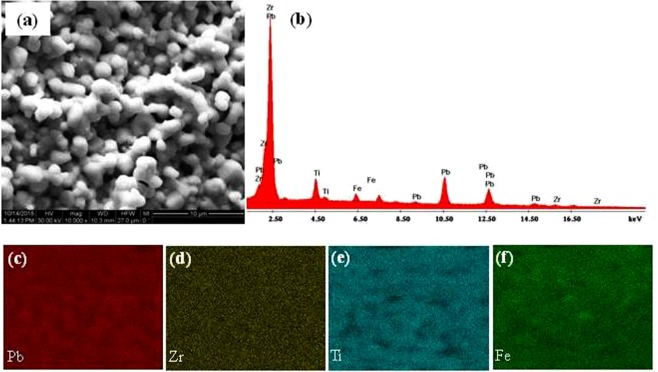
Characterization of microstructure and homogeneity of sintered PZT-5%Fe_3_O_4_. (**a**–**f**) SEM data for a sintered PZT-5%Fe_3_O_4_ composite in its as-prepared form (without polishing). (**a**) SEI topography image with magnification x10000. (**b**) BSE-based EDS spectrum for elemental analysis. (**c**–**f**) BSE-based EDS compositional mapping referring to (**c**) Pb, (**d**) Zr, (**e**) Ti, and (**f**) Fe.

Figure 4(a–f) present detailed SEM data referring to, topography (Fig. 4(a)), EDS spectrum for elemental analysis (Fig. 4(b)), and EDS elemental mapping (Fig. 4(c–f)) for a sintered PZT-5%Fe_3_O_4_ composite (sintering conditions: T = 1000 °C, for t = 2 h in air) in its as-prepared form (without polishing). These results prove that the sample is compact with the expected porosity, while all elements are uniformly distributed fairly well (we note that some inhomogeneity is expected since the composition of the sample is at the MPB). The respective data referring to a sintered PZT-5%Fe_3_O_4_ composite (sintering conditions: T = 1000 °C, for t = 2 h in air) after polishing are presented in Supplementary Information (see ‘Section II’).

**Figure 5:**
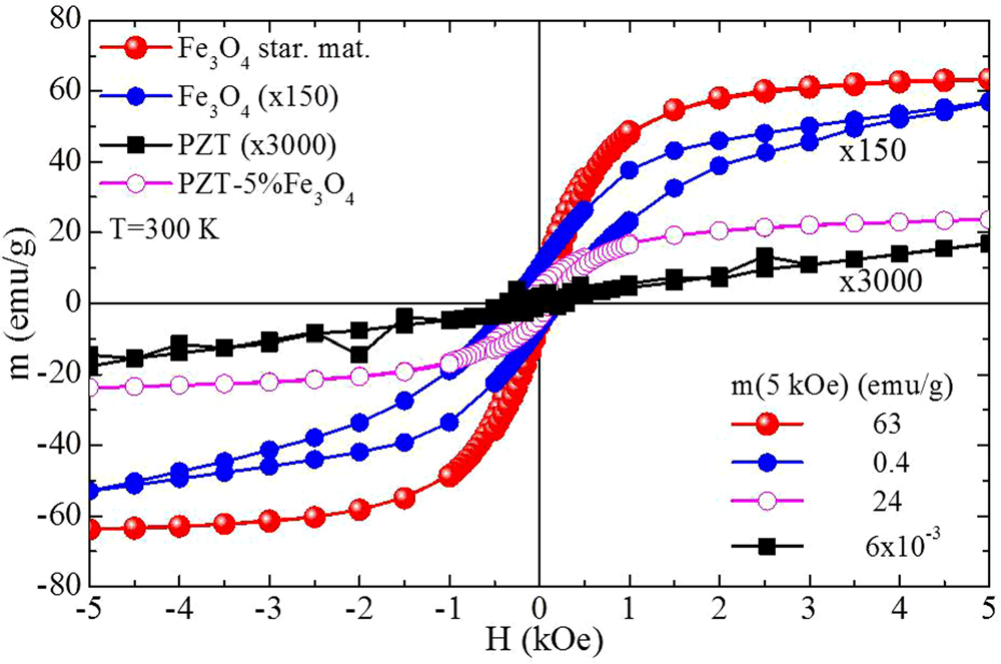
Magnetization data for non-sintered Fe_3_O_4_ NPs, sintered Fe_3_O_4_ NPs, sintered PZT, and sintered PZT-5%Fe_3_O_4_ samples. Magnetization curves, m(H), obtained in non-sintered Fe_3_O_4_ NPs (starting material: star. mat.), sintered Fe_3_O_4_ NPs, sintered PZT, and sintered PZT-5%Fe_3_O_4_ samples for an external magnetic field up to 5 kOe. The curve of sintered Fe_3_O_4_ NPs and sintered PZT is multiplied by a factor of 150 and 3000, respectively, for the sake of presentation. The magnetization value, m(5 kOe), obtained at the maximum applied magnetic field, 5 kOe, for each sample reads 63 emu/g for non-sintered Fe_3_O_4_ NPs, 0.4 emu/g for sintered Fe_3_O_4_ NPs, 6 × 10^−3^ emu/g for sintered PZT, and 24 emu/g for the sintered PZT-5%Fe_3_O_4_ composite.

The magnetization data of Fig. 5 accompany the XRD results and further support the conclusions discussed above. Specifically, Fig. 5 presents m(H) data for non-sintered Fe_3_O_4_ NPs (starting material: star. mat.), sintered Fe_3_O_4_ NPs, sintered PZT, and the sintered PZT-5%Fe_3_O_4_ composite (sintering conditions: T = 1000 °C, for t = 2 h in air). The deduced magnetization values for a magnetic field of 5 kOe are m = 63 emu/g, 0.4 emu/g, 6 × 10^−3^ emu/g, and 24 emu/g, respectively. We see that the non-sintered Fe_3_O_4_ NPs have high magnetization value, 63 emu/g, however lower than that of bulk magnetite due to their reduced size. When the Fe_3_O_4_ NPs are sintered in free form (not included in a PZT matrix) they completely oxidize to α-Fe_2_O_3_ as evidenced by the extremely lower magnetization value, 0.4 emu/g, obtained after sintering that is the magnetic fingerprint of hematite^44^. Referring to sintered PZT, it exhibits negligible magnetization, 6 × 10^−3^ emu/g, as expected. Accordingly, we can conclude that for the sintered PZT-5%Fe_3_O_4_ composite the deduced magnetization value, 24 emu/g, originates from Fe_3_O_4_ NPs that have not oxidized to α-Fe_2_O_3_ since they are protected by the PZT matrix. Using these magnetization values we can estimate that 38% of the NPs remain in the Fe_3_O_4_ form after sintering. In agreement to the XRD results the magnetization data show that the former PZT-5%Fe_3_O_4_ system evolves to a PZT-5%(Fe_3_O_4_/α-Fe_2_O_3_) composite (for clarity, simply denoted PZT-5%Fe_3_O_4_ in the rest of the paper).

**Figure 6:**
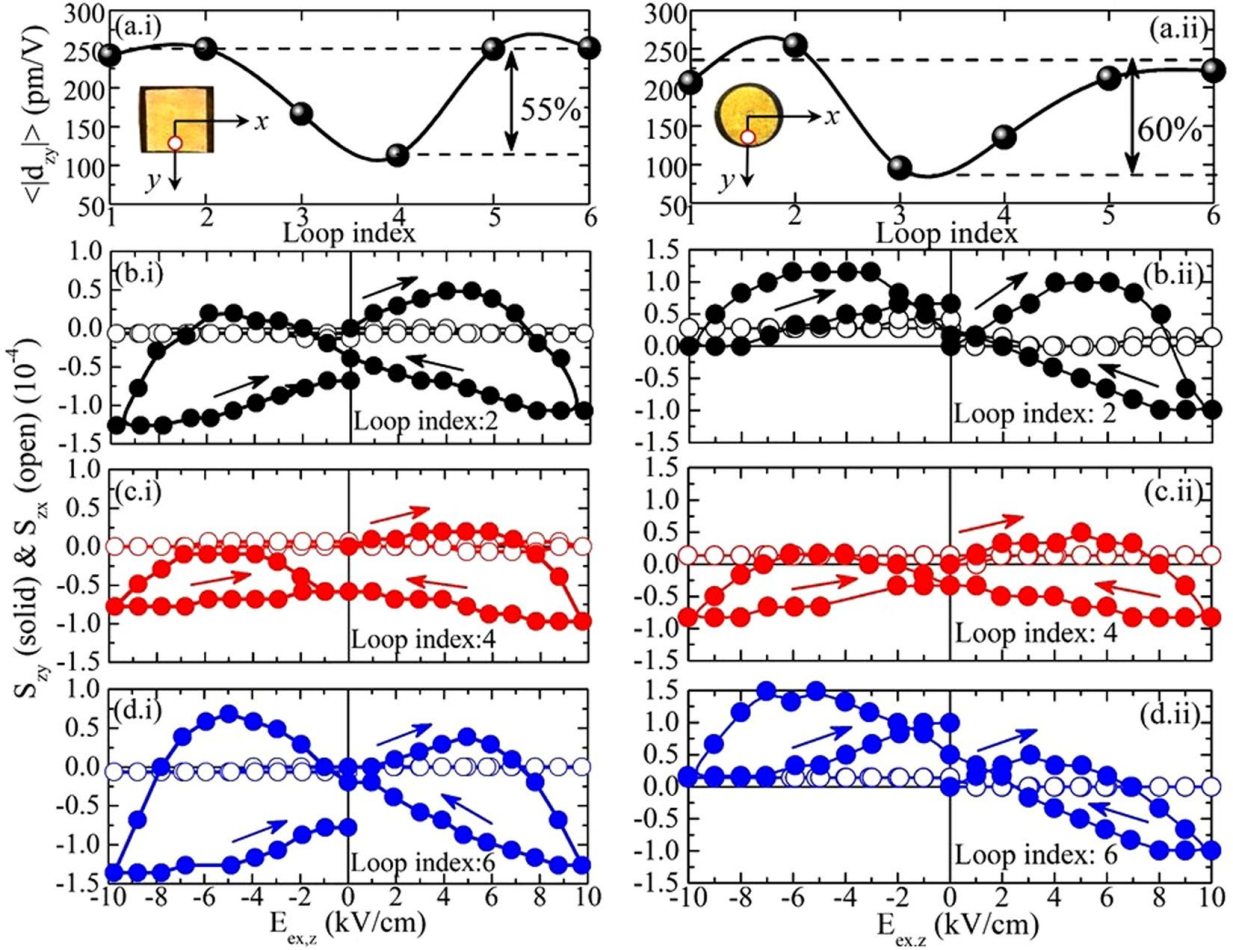
Piezoelectric data for PZT-5%Fe_3_O_4_ samples in the absence/presence of a constant out-of-plane magnetic field, H_ex,z_. (**a**.**i**–**d**.**i**) & (**a**.**ii**–**d**.**ii**) Piezoelectric data for (**a**.**i**–**d**.**i**) square-shaped (length 7 mm, thickness 0.4 mm) and (**a**.**ii**–**d**.**ii**) disc-shaped (diameter 6 mm, thickness 0.4 mm), PZT-5%Fe_3_O_4_ samples. (**a**.**i**,**a**.**ii**) Mean value of PE coefficient originating for each case from the absolute values of the entire S_zy_(E_ex,z_) loop, < |d_zy_| > , as function of H_ex,z_ = 1 kOe; in the absence of H_ex,z_ (Loop index: 1 & 2), under application of H_ex,z_ (Loop index: 3 & 4) and after the removal of H_ex,z_ (Loop index: 5 & 6). The inset photos show the respective samples together with the arbitrarily chosen x and y coordinate system. The investigated area is shown with a dot placed on SA_y_ for both samples. (**b**.**i**–**d**.**i**)/(**b**.**ii**–**d**.**ii**) S_zy_(E_ex,z_) and S_zx_(E_ex,z_) loops, solid and open points respectively, for the square/disc-shaped sample upon application and removal of H_ex,z_ = 1 kOe; (b.i)/(b.ii) in the absence of H_ex,z_ (Loop index: 2), (**c**.**i**)/(**c**.**ii**) under application of H_ex,z_ (Loop index 4) and (**d**.**i**)/(**d**.**ii**) after the removal of H_ex,z_ (Loop index: 6). The arrows trace the sequence of E_ex,z_ application. Please, notice that the vertical scale is the same in each group of three panels, (**b**.**i**–**d**.**i**) and (**b**.**ii**–**d**.**ii**). The raw data of panels (**c**.**i**,**d**.**i**) are accessible in ‘Section III’ of Supplementary Information and the accompanying Supplementary Videos.

Figure 6 shows detailed data of the in-plane PE coefficients obtained upon application and removal of a constant H_ex,z_ for a square-shaped sample, (a.i)–(d.i), and for a disc-shaped sample, (a.ii)–(d.ii). The insets of the upper panels, (a.i) and (a.ii), show photos of the specific samples together with an arbitrarily chosen coordinate system that assists us to define the symmetry axes x (SA_x_) and y (SA_y_). As it should be, the investigated area was at the sample edge, in the specific case onto SA_y_, as shown with a white dot for both samples (for details see ‘Section III’ of Supplementary Information and^37,38^). Concerning the experimental procedure, six consecutive E_ex,z_ loops were recorded designated by a loop index; first, without the application of H_ex,z_ (loop index: 1 and 2), next, under the presence of H_ex,z_ = 1 kOe (loop index: 3 and 4) and finally, when removing H_ex,z_ (loop index: 5 and 6). Figure 6(a.i,a.ii) present the mean absolute value of the in-plane PE coefficient in the y-direction, <|d_zy_|>, for the complete sequence of measurements (the other in-plane PE coefficient in the x-direction, d_zx_, is not shown since it is obviously zero^37,38^). Specifically, these panels present explicit data on < |d_zy_| > before applying (loop index: 1 and 2), upon application (loop index: 3 and 4) and after removal (loop index: 5 and 6) of a constant H_ex,z_ = 1 kOe. For each loop the <|d_zy_|> is calculated from the slopes |dS_zy_/dE_ex,z_| of the linear parts of the detailed S_zy_(E_ex,z_) data. Representative sets of the latter are shown in Fig. 6(b.i–d.i,b.ii–d.ii) for the square-shaped and disc-shaped sample, respectively, for loop index: 2 (without H_ex,z_, (b.i) and (b.ii)), loop index: 4 (with H_ex,z_, (c.i) and (c.ii)) and loop index: 6 (without H_ex,z_, (d.i) and (d.ii)). The raw data of panels (c.i) and (d.i) are accessible in ‘Section III’ of Supplementary Information and the accompanying Supplementary Videos. Please note that, as expected^37,38^, the respective S_zx_(E_ex,z_) loops are practically horizontal lines, since the investigated area was onto SA_y_ (see insets of Fig. 6(b.i–b.ii)). With the help of the raw data presented in Fig. 6(b.i–d.i)/(b.ii–d.ii), the main results of the present work become evident: first, the S_zy_(E_ex,z_) curves become narrower upon H_ex,z_ application (Fig. 6(b.i)/(b.ii) vs Fig. 6(c.i)/(c.ii)), and second they recover their original form, almost completely, upon H_ex,z_ removal (Fig. 6(b.i)/(b.ii) vs Fig. 6(d.i)/(d.ii)). Thus, this data evidences that the application of an external magnetic field, H_ex,z_ = 1 kOe, causes drastic decrease to the respective in-plane PE coefficients on the order of 50–60% which is restored upon H_ex,z_ removal, as summarized in the deduced <|d_zy_|> data that are presented in Fig. 6(a.i)/(a.ii).

**Figure 7:**
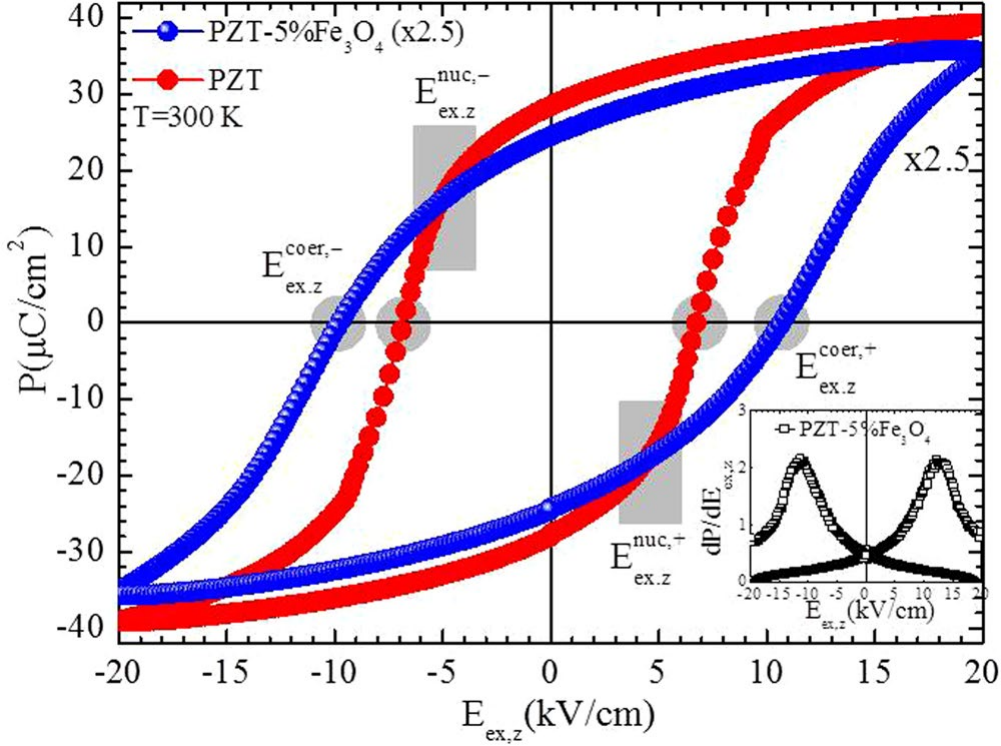
Polarization data for both PZT and PZT-5%Fe_3_O_4_ samples. Polarization curves, P(E_ex,z_), for both PZT and PZT-5%Fe_3_O_4_ samples for an external electric field along the z axis, E_ex,z_, up to 20 kV/cm. The curve of the PZT-5%Fe_3_O_4_ sample is multiplied by a factor of 2.5 for the sake of presentation. The nucleation fields, E_ex,z_^nuc,+^ and E_ex,z_^nuc,−^ and coercive fields, E_ex,z_^nuc,+^ and E_ex,z_^nuc,−^ are marked with the gray-shaded areas. Inset presents the derivative, dP/dE_ex,z_, for the case of the PZT-5%Fe_3_O_4_ composite.

To clarify the S_zy_(E_ex,z_) curves obtained with our local OM-based method we performed standard measurements of the polarization in both sintered PZT and PZT-5%Fe_3_O_4_ (sintering conditions: T = 1000 °C, for t = 2 h in air). Figure 7 shows raw P(E_ex,z_) data, while its inset presents the derivative, dP/dE_ex,z_, for the case of the PZT-5%Fe_3_O_4_ composite. From this data becomes apparent that the addition of the FM Fe_3_O_4_ NPs to PZT makes it harder; the P(E_ex,z_) loop is noticeably broadened since the coercive field, E_ex,z_^coer^, increases from 6.7 kV/cm to 10.7 kV/cm. This fact can be ascribed to the efficient pinning of FE domain walls by the structural disorder introduced by the FM NPs; the similar size (50–100 nm) of the FM Fe_3_O_4_ NPs (see ‘Sample preparation’ in ‘METHODS’) and the FE domains of PZT (see^17,39–41^) can probably motivate and or promote this feature. On the other hand, the nucleation field, E_ex,z_^nuc^, is practically unaltered at approximately 4–5 kV/cm. Notably, the P(E_ex,z_) data clearly show that the peaks observed in the S_zy_(E_ex,z_) curves of Fig. 6(b.i–d.i)/(b.ii–d.ii) correspond to the nucleation fields, E_ex,z_^nuc,+^ and E_ex,z_^nuc,−^, where FE domains appear and start to move/rotate, ultimately dictating complete reversal of the bulk polarization. Most important, the comparison of the S_zy_(E_ex,z_) results with the P(E_ex,z_) ones clearly evidences that the FM Fe_3_O_4_/Fe_2_O_3_ NPs do not simply serve as *static structural disorder*; they introduce *reconfigurable magnetic disorder* that modifies the in-plane strain-electric field curves and the accompanying PE coefficients when an external magnetic field is applied at will.

Referring to the underlying mechanism motivating the observed ME coupling between the PZT matrix and the Fe_3_O_4_ NPs we recall that three mechanisms are generally considered: first charge modulation, second exchange interaction modulation and third strain transfer^45^. Since the first two mechanisms are active at a short range by a FE/FM interface, we suggest that the strain transfer mechanism is dominant in the case of the PE/FM PZT-5%Fe_3_O_4_ samples studied here (noticeably, the latter is active at length scales on the order of 100–200 nm^46,47^). Recently, A. Kumar *et al*.^48^ employed the strain transfer mechanism to explain the results obtained in a relevant multiferroic BiFeO_3_-BaTiO_3_ alloy system in which FM arises from a small fraction (~1 wt%) of BaFe_12_O_19_ that is formed during sintering. Interestingly, a very large increase (~34%) of the saturation polarization was observed under application of a magnetic field 10 kOe. The authors^48^ attributed this finding to transfer of magnetostrictive strain from the FM BaFe_12_O_19_ grains to the adjacent ones of the FE phase. Specifically, since the magnetostriction coefficient, λ, of BaFe_12_O_19_ is negative (~ −1.5 × 10^−5^) it was suggested that the contraction of the FM grains makes the FE ones to expand so that the pinning potential of the respective domain walls seemed to effectively reduce, ultimately enhancing their mobility^48^. In our case the same mechanism could be at play due to the strong PE character of PZT and the noticeable magnetostrictive nature of Fe_3_O_4_ and Fe_2_O_3_. Specifically, the magnetostriction coefficient, λ, of bulk magnetite ranges within 20 × 10^−6^ to 90 × 10^−6^ for single crystals and 30 × 10^−6^ to 50 × 10^−6^ for polycrystalline samples^49,50^. In addition, the magnetostriction coefficient of single crystals of bulk hematite is somehow lower, 10 × 10^−6^ at maximum^51^. For the case of NPs of Fe_3_O_4_ and Fe_2_O_3_ these values do not change except when considering ultra small ones with size 5 nm^52^. We see that the magnetostriction coefficient values of Fe_3_O_4_ and Fe_2_O_3_ are almost comparable to the PE coefficient ones of PZT (10^−4^). Thus, we can assume that an effective interface strain transfer is realized between the Fe_3_O_4_ and Fe_2_O_3_ NPs (especially the Fe_3_O_4_ ones) and the surrounding PZT matrix. In this context, the magnetostrictive strain of Fe_3_O_4_ and Fe_2_O_3_ NPs (especially the Fe_3_O_4_ ones) can effectively alter the PE strain of the PZT matrix when an external magnetic field is applied. Indeed, the experimental fact that the PE coefficients are completely restored upon removal of the magnetic field (reversible procedure) hints that the underlying cause is a linear magnetostrictive effect of magnetoelastic nature.

Irrespectively of the underlying mechanism, here we report for the first time experimental data on the magnetic-field control of a ME parameter, specifically that of the PE coefficients d_zi_ (i = x,y), of three significant characteristics: first, it is impressive in magnitude, 60%, second, it is completely reversible, while third, and probably most important, these characteristics are feasible at room temperature upon using a relatively low magnetic field, H_ex_ = 1 kOe. At this point, it is worth mentioning that recent studies^35,36^ concerning composite ME systems report variations of similar magnitude in other relevant parameters such as the polarization and piezoresponse, but not in the PE coefficients reported here. In^35^ D. M. Evans *et al*. report an average change of 60% in the polarization of PZTFT single crystals for a relatively high H_ex_ variation of 6 kOe, between the +3 kOe and −3 kOe states. Unfortunately, as already discussed by the authors^35^, this noticeable percentage variation is partially reversible upon reversing once again the magnetic field back to +3 kOe, since the polarization attains a 40% higher value in comparison to that of the original state. In^36^ S. H. Xie *et al*. observe a change of 40% in the piezoresponse of PZT-TDF bilayer upon application of +2 kOe. Notably, in contrast to the partially reversible behavior observed in^35^, in^36^ the authors report a further increase of the piezoresponse upon reversing the magnetic field to −2 kOe, a clear proof that in this case the overall process is entirely irreversible. The above comparative discussion with the data reported in^35,36^ shout that our results could be important for applications at room temperature.

## Conclusions

Here, we investigated the modulation of PE coefficients for a bulk PZT-5%Fe_3_O_4_ system, which is a strongly insulating hybrid of FM Fe_3_O_4_ NPs (noticeably magnetostrictive) homogeneously embedded in a FE PZT matrix (highly piezoelectric). By means of an OM-based method we recorded the in-plane strain loops for electric fields applied out-of-plane in the range −10 kV/cm ≤ E_ex,z_ ≤+ 10 kV/cm, upon successive application and removal of an also out-of-plane external magnetic field of low value, H_ex,z_ = 1 kOe. The respective in-plane PE coefficients, 200–250 pm/V, display a dramatic decrease on the order of 50–60% upon H_ex,z_ application, that is completely restored upon H_ex,z_ removal. Polarization data performed in a wider range −20 kV/cm ≤ E_ex,z_ ≤ + 20 kV/cm showed that the addition of the FM Fe_3_O_4_ NPs makes the FE PZT harder obviously due to the pinning of FE domain walls by the newly introduced *static structural disorder*. The combined results clearly evidence that the FM Fe_3_O_4_ NPs do not simply serve as *static structural disorder*; they introduce *reconfigurable magnetic disorder* that modifies the in-plane strain-electric field curves and the accompanying PE coefficients when an external magnetic field is applied at will. Based on the strong PE character of PZT and the noticeable magnetostrictive nature of Fe_3_O_4_, these findings can be explained by a strain transfer mechanism realized at the interface of the FM Fe_3_O_4_ NPs and the surrounding FE PZT matrix. The high and entirely reversible modulation of the PE coefficients, under the application and removal of a low magnetic field, as well as the room temperature feasibility of the above advantages, render the PZT-x%Fe_3_O_4_ composite system or similar ones promising candidates for applications.

## Methods

### Sample Preparation

The starting materials were Pb(Zr_0.52_Ti_48_)O_3_ powder, PZT5H type simply called PZT (NCE55, Noliac) and Fe_3_O_4_ (Sigma-Aldrich 98%, particle size: 50–100 nm). Fe_3_O_4_ powder in concentration x = 0–50% per weight, was grounded and mixed thoroughly with PZT powder into an agate mortar, in order to achieve homogeneous hybrid oxides. The mixed oxides were pressed into pellets at 35 MPa, with diameter 20 mm, placed on alumina crucibles and sintered at T_sin_ = 1000 °C, for t = 2 h, in air. The heating (≈100 °C/h) and cooling (≈50 °C/h) rates of the furnace (Carbolite Co. Ltd, UK) were kept constant for all the samples prepared. Here, we investigated samples of different shapes and dimensions. Specifically, the sintered PZT-x%Fe_3_O_4_ were cut at square-shaped sample with dimensions 7 × 7 mm^2^ and disc shaped sample with diameter 6 mm. In order to apply external electric fields as high as E_ex,z_ = 10 kV/cm, we reduced the thickness of the samples, t, from t = 1 mm down to t = 0.4 mm. To this end, the surfaces of the samples were polished using sandpaper, rinsed with ethanol and air dried. For E_ex,z_ application, Au films (~100 nm) were sputtered on both surfaces of the samples (E5100, Quorum Technologies Ltd, Eats Sussex, UK), which also serve as reflective surfaces for observation with OM. During sputtering a macroscopic mask was employed to protect the lateral surfaces of the sample and avoid any short circuit while applying the external voltage. The samples were left unpoled.

### Techniques

Since the present study aims to attain information that could be of interest for practical applications it is exclusively focused at room temperature. As a consequence, all experimental techniques described below were employed at room temperature.

#### X-ray diffraction (XRD)

The compositional and structural characterization of Pb(Z_0.52_T_0.48_)O_3_, Fe_3_O_4_ and the hybrid composite Pb(Zr_0.52_Ti_0.48_)O_3_-5%Fe_3_O_4_ was obtained by means of XRD measurements performed with an X-ray diffractometer (D500, Siemens) that employs coupled θ–2θ scans with CuKα (wavelength λ = 1.5418 Å) as the radiation source.

#### Scanning Electron Microscopy (SEM)

SEM images were obtained by means of Inspect Microscope (FEI, Hillsboro, OR, USA) working with W (tungsten) filament. Regarding sample preparation, when necessary, PZT-5%Fe_3_O_4_ samples were sputtered with a thin layer (20–50 nm) of Au under medium vacuum (10^−1^ Torr) by means of a typical sputtering unit (E5100, Quorum Technologies Ltd, Eats Sussex, UK). Then the sample was placed onto conventional pin stubs. During SEM we employed typical values for the interfering parameters (i.e. acceleration voltage within 15–30 kV, working distance within 8–15 mm and spot size 3–8). Information on the topography for the evaluation of the microstructure was obtained with secondary electron imaging (SEI), while elemental analysis to obtain both energy-dispersive x-ray spectroscopy (EDS) information and compositional mapping images was recorded with backscattered electron imaging (BSE). Au-coated samples were used for SEI, while both Au-coated and non-coated samples were used for BSE to cross-check the results due to the overlapping of the M-spectral line of Au (2.123 keV) with the Lα-spectral line of Zr (2.044 keV) and the M-spectral line of Pb (2.342 keV).

#### Current-voltage (I–V) characteristics

I–V characteristics were taken for the composite Pb(Zr_0.52_Ti_0.48_)O_3_-x%Fe_3_O_4_ samples, x = 0–50%, using a DC-voltage supply (model IP-32, Healthkit Co., USA) to apply the voltage across each sample, while *I* was monitored with the digital multimeter (MY-67, V&A). This information is important since we want to apply the maximum possible electric field to the sample without loss of its insulating properties.

#### Optical Microscopy for piezoelectric characterization

The constitutive Strain-Electric field curve, S(E_ex,z_), of the samples was estimated experimentally by using an OM-based technique introduced in^37^. An ORTHOLUX (Leitz, Wetzlar, Germany) OM was used, equipped with a linear xy translation stage on which a home-made aluminum platform was mounted. The magnification used was x100-x150 (objective lens x10). The calibration in the length scale of the OM images was accomplished by using the standard grating test TGZ3 (NT-MDT Co, Moscow, Russia). Using a DC-voltage supply (model IP-32, Healthkit Co., USA) we applied E_ex,z_ up to 10 kV/cm along the sample thickness, i.e. out-of-plane, during the measurements. More details can be found in^38^. Here, we made another modification in the home-made aluminum platform hosting the sample, so that a constant magnetic field, H_ex,z_, is applied at will, also out-of-plane (z axis), by a NdFeB permanent magnet, (disc-shaped with diameter 20 mm and thickness 3 mm). Mapping of H_ex,z_ was performed with a Gaussmeter Model 410 (Lake Shore Cryotronics Inc, Ohio, USA), with its probe placed on a linear xyz stage with micrometer resolution.

#### Magnetization measurements

The constitutive Magnetization-Magnetic field curve, M(H_ex,z_), of the samples was measured experimentally by using a SQUID magnetometer MPMS 5.5 Tesla (Quantum Design, San Diego, CA, USA).

#### Polarization measurements

The constitutive Polarization-Electric field curve, P(E_ex,z_), of the samples was measured experimentally by using a TF Analyzer 2000 (aixACCT Systems GmbH, Aachen, Germany) ferroelectric analyzer connected with a high-voltage source Trek Model 610E (TREK INC, Lockport, NY, USA). The samples were immersed in silicone oil during the measurements to prevent arcing. The waveform and frequency of the applied electric field are triangle and 10 Hz, respectively.

## Supplementary information

Supplementary Information

SV1-Vertsioti-Zhang-Stamopoulos

SV2-Vertsioti-Zhang-Stamopoulos
