# Supplementary material for: Pronounced and reversible modulation of the piezoelectric coefficients by a low magnetic field in a magnetoelectric PZT-5%Fe3O4 system
Source: Sci Rep. 2019 Feb 18;9:2178. doi: 10.1038/s41598-019-38675-8 (PMC6379417; doi:10.1038/s41598-019-38675-8)
Supplement: Supplementary file 1 — Supplementary Information [file 41598_2019_38675_MOESM1_ESM.pdf]

Supplementary Information

Pronounced and reversible modulation of the piezoelectric coefficients by a low magnetic field in a magnetoelectric PZT-5%Fe_3O_4 system

G. Vertsioti[1,2], S. J. Zhang[3], and D. Stamopoulos[1,2,]*
[1]Department of Solid State Physics, National and Kapodistrian University of Athens, Zografou Panepistimioupolis, Athens, Greece.
[2]Institute of Nanoscience and Nanotechnology, National Center for Scientific Research 'Demokritos', Aghia Paraskevi, Athens, Greece.
[3]Institute for Superconducting and Electronic Materials, Australian Institute of Innovative Materials, University of Wollongong, Australia.

***Corresponding author: Prof. Dr. Dimosthenis Stamopoulos
Department of Solid State Physics, National and Kapodistrian University of Athens, Zografou Panepistimioupolis, Athens, Greece
Phone: +30-210-7276823; Fax: +30-2107276711
E-mail: d.stamopoulos@inn.demokritos.gr & densta@phys.uoa.gr

Section I: Rejection of in-plane shift caused by any possible drift between mechanical parts of the experimental setup

In any standard commercially available OM possible shifts due to undesired drift between its mechanical parts (e.g. optical stage and head), either over the x-y plane (in-plane) or along the z axis, are practically zero. In our case, another component has been added onto the stage of the OM, namely the sample-carrying platform, Supplementary Figure 1. This extra part is not tightly fixed onto the stage; it is mounted to an in-plane slider by an adjustment clip. It is easy to understand that the sample-carrying platform is vulnerable to possible drift relatively to both the in-plane slider and stage over the x-y plane, at least at a transient time period where mechanical relaxation exists. This could be a serious source of error, since in our case we record the in-plane deformation of the sample that ultimately enables us to calculate the respective $S_{zx}(E_{ex,z})$ and $S_{zy}(E_{ex,z})$ curves. To eliminate such errors, we carefully monitored the in-plane shift along both the x and y axes to identify the time limit of the transient state, after which any possible mechanical drift safely ceases, Supplementary Figures 2(a) and 2(b), respectively. From these measurements we concluded that the transient state lasts for $t \approx 80$ min at maximum. Accordingly, in all our experiments where the sample-carrying platform had to be removed and repositioned onto the stage of the OM (e.g. at the first measurement or when the NdFeB permanent magnet had to be used) we waited for almost 2 hours before starting our measurements.

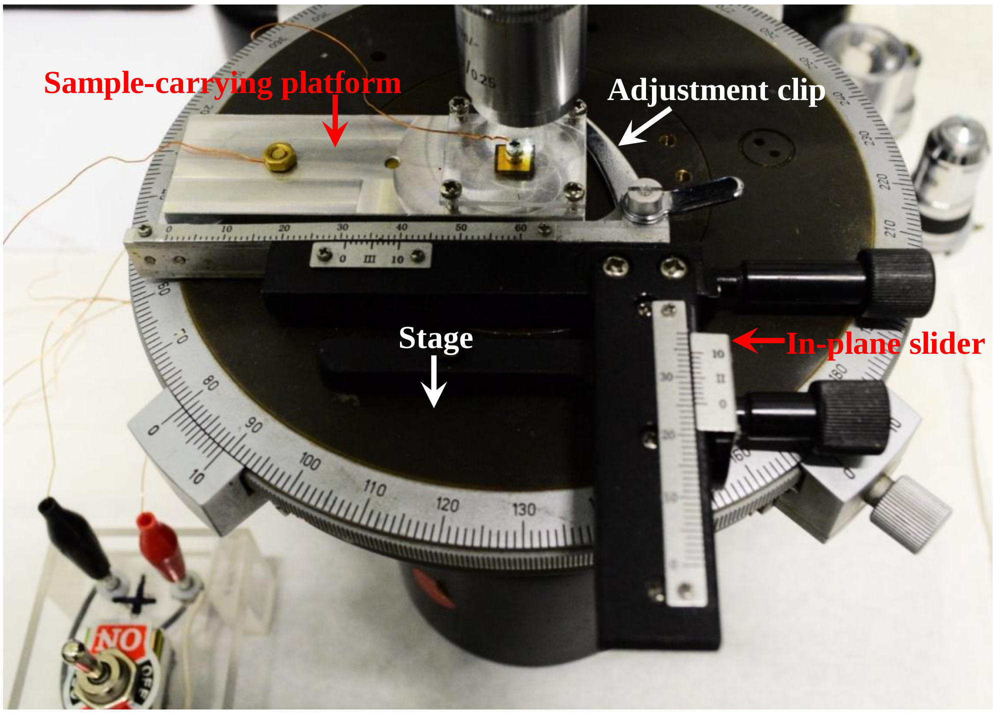

Supplementary Figure 1: Perspective view of OM stage upon recording the in-plane deformations of the sample, in order to calculate $S_{zx}(E_{ex,z})$ and $S_{zy}(E_{ex,z})$. The sample-carrying platform is mounted to a fixed in-plane slider by an adjustment clip.

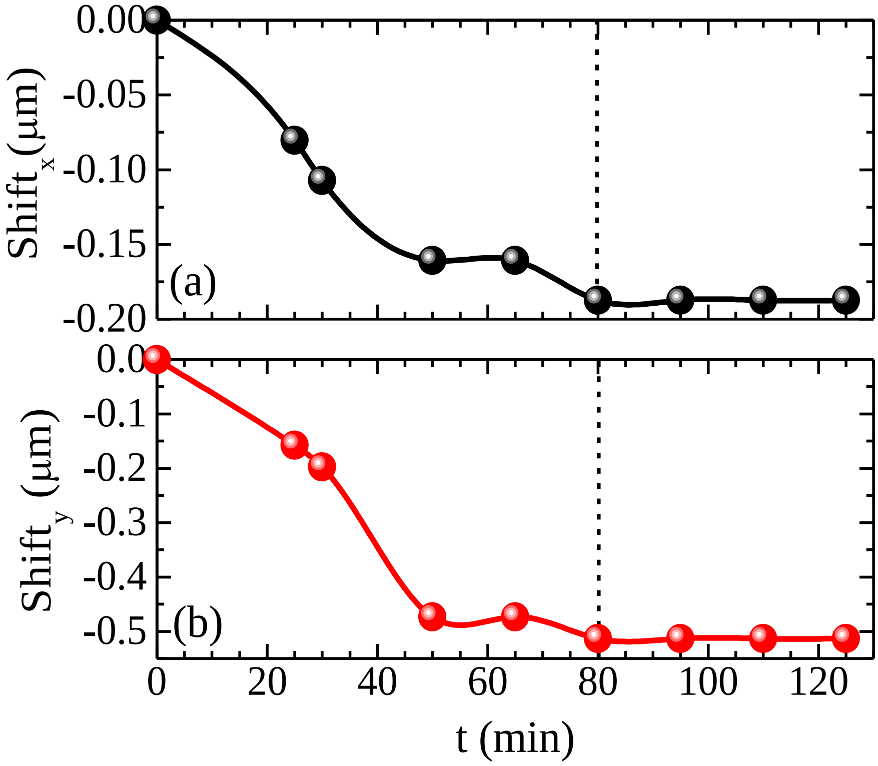

Supplementary Figure 2: Measurements of the in-plane shift along (a) x and (b) y axes caused by any possible drift between mechanical parts of the experimental setup e.g. the sample-carrying platform and optical stage. After adequate time, t≈80 min, the in-plane shift along both x and y axes is practically zero.

Section II: Evaluation of homogeneity in the bulk of sintered PZT-5%Fe_3O_4

Figures 3(a)-3(g) present detailed SEM data referring to, topography (Fig. 3(a)), EDS spectrum for elemental analysis (Fig. 3(b)), and EDS elemental mapping (Figs. 3(c)-3(g)) for a sintered PZT-5%Fe_3O_4 composite (sintering conditions: T=1000 ^{0}C, for t=2 h in air) in its polished and Au-coated form. These results prove that in the bulk of the sample, all Pb, Zr, Ti, and Fe are uniformly distributed fairly well (we note that some inhomogeneity is expected since the composition of the sample is at the MPB). Data referring to the surface of a sintered PZT-5%Fe_3O_4 composite in its as-prepared form (without polishing and Au coating) are presented in Figs. 4(a)-4(f) of the paper.

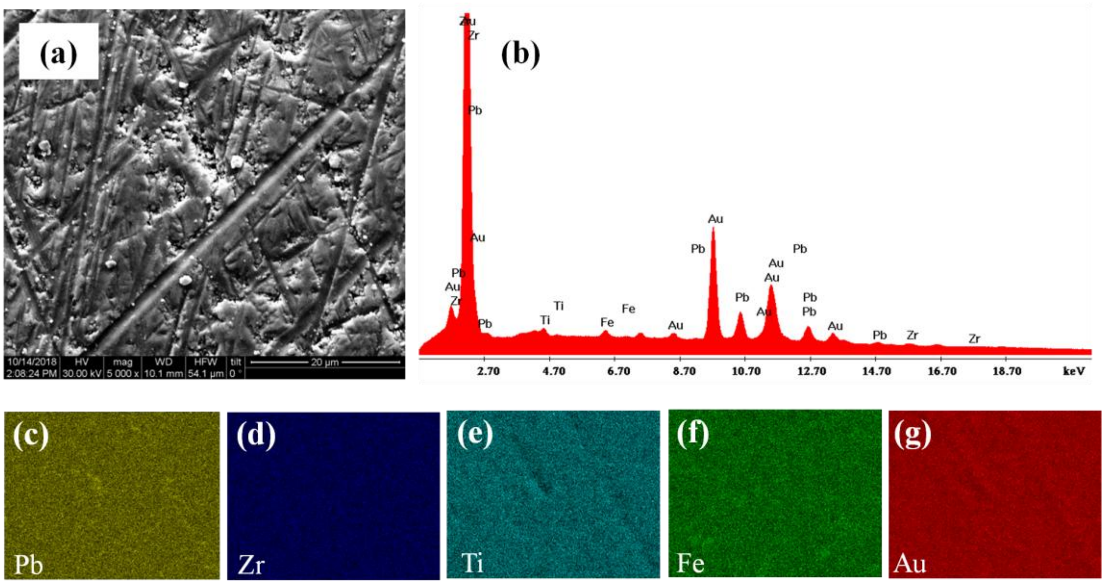

Supplementary Figure 3: (a)-(g) SEM data for a sintered PZT-5%Fe_3O_4 composite after polishing and Au coating. **(a)** SEI topography image with magnification x5000. **(b)** BSE-based EDS spectrum for elemental analysis. **(c)-(g)** BSE-based EDS compositional mapping referring to **(c)** Pb, **(d)** Zr, **(e)** Ti, **(f)** Fe and **(g)** Au.

Section III: Demonstration of the experimental procedure and quantitative clarification of the obtained in-plane $S_{zx}(E_{ex,z})$ and $S_{zy}(E_{ex,z})$ data

For a polycrystalline sample (without any crystallographic texture/preferred orientation) the strain/piezoelectric coefficients recorded at different sides/points of a rectangle/disc sample should be the same. This is why in the text we employ the terms "in-plane strain" and "in-plane piezoelectric coefficients", without distinguishing the crystallographic axes a, b and c. Thus, the employed axes 'x' and 'y' (for instance, in the insets of Figs. 6(a.i) and 6(a.ii) of the paper) are used to simply denote a (guide to the eye) coordinate system that illustrates in which specific area of each sample the measurements were recorded. This information is important since it proves the reliability of the employed procedure; the deformation of a polycrystalline rectangle sample (without crystallographic texture/preferred orientation) recorded on an axis placed at the middle of one of its side should be exclusively along the direction of this specific axis (in this specific axis, let us say 'y', the sample should not exhibit any deformation along the vertical direction, let us say 'x', else the sample would rotate).

In the same spirit, the response recorded along two vertical symmetry axes placed at the middle of the two rectangle-sample's sides should be the same. Indeed, this is what we observe. To clarify this issue we present additional data in Supplementary Figure 4, below. This data is obtained in a rectangle sample, of different side lengths 3 mm and 6 mm, *when the observation point is located at its down left corner*, as illustrated in the inset of panel (a). Thus, in this case we should observe strain in both directions that should be of the same magnitude. Indeed, this is the case observed in the data presented in panel (a) (referring to strain along the 'x' axis, $S_{zx}(E_{ex,z})$) and in panel (b) (referring to strain along the 'y' axis, $S_{zy}(E_{ex,z})$).

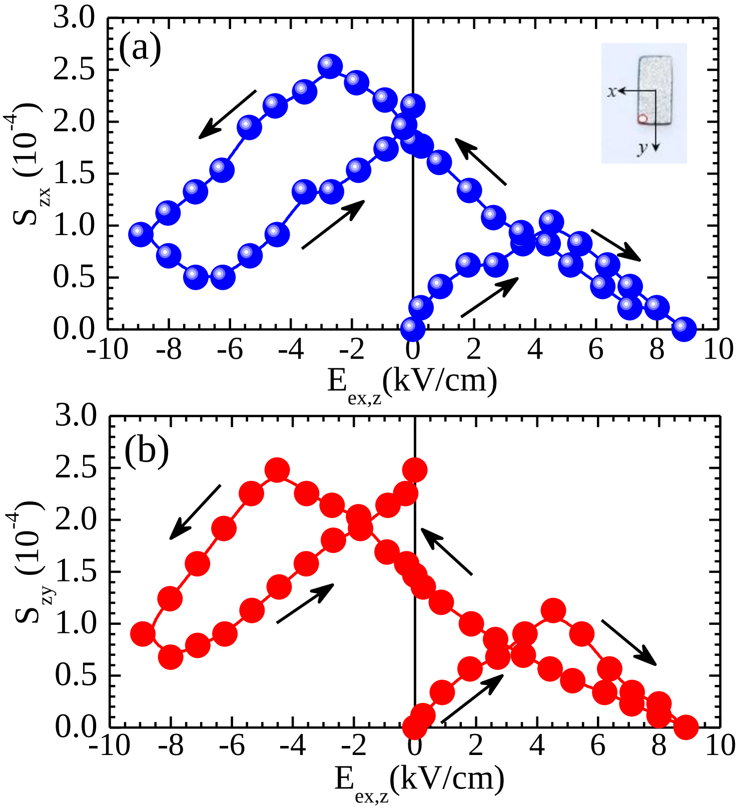

Supplementary Figure 4: Measurements of the in-plane Strain along (a) the 'x' axis and (b) the 'y' axis for a rectangle sample with sides' length 3 mm and 6 mm, *when the observation point is located at its down left corner*, as illustrated in panel (a). Strain of the same magnitude is observed in both vertical directions as expected.

Additional evidence is provided by the two supplementary videos, SV1_Vertsioti-Zhang-Stamopoulos [1] referring to loop index 4 (Fig. 6 (c.i) of the paper) and SV2_Vertsioti-Zhang-Stamopoulos [2] referring to loop index 6 (Fig. 6 (d.i) of the paper). These supplementary videos present a sequence of photographs obtained with optical microscope while varying $E_{ex,z}$ within ±10 kV/cm, before (SV1_Vertsioti-Zhang-Stamopoulos) and after (SV2_Vertsioti-Zhang-Stamopoulos) the application of an $H_{ex,z}$=1 kOe. Panel (a) presents the sample and designates the imaged area chosen along a specific symmetry axis (y-axis in the specific case). Panel (b) shows the complete $S_{zx}(E_{ex,z})$ and $S_{zy}(E_{ex,z})$ data referring to the x and y axis, respectively (Fig. 6 (c.i) and 6 (d.i) of the paper reproduced here for the sake of clarity). Finally, panel (c) focuses on the imaged area and presents the initial position of the characteristic point (CP) with an extended dashed cross. The center of the latter is fixed and serves as the

reference point of the initial unstrained state ($E_{ex,z}$=0). On the contrary, the limited solid cross traces the position of the CP as we vary $E_{ex,z}$ in the following sequence of photographs. Thus, the resulting displacement can be quantified by means of the scale-bar that designates 1 μm. The specific value of $E_{ex,z}$ is shown in the upper left corner of each photograph, while the deduced $S_{zx}(E_{ex,z})$ and $S_{zy}(E_{ex,z})$ values are shown in panel (b). By careful observation of these videos we deduce that for both cases the displacement along x-axis is at the borderline of the resolution limit of standard optical microscopy (200 nm), while the ones along the y-axis are well above. Consequently, we completely ignore displacements along x-axis so that almost all data points referring to the respective $S_{zx}(E_{ex,z})$, presented in panel (b) of each slide, are placed to zero. In contrast, $S_{zy}(E_{ex,z})$ obtains clearly non-zero values, denoted by the green circle that traces the entire $S_{zy}(E_{ex,z})$ loop in panel (b). For more details on the overall experimental procedure see [37,38] of the paper.

[1] SV1_Vertsioti-Zhang-Stamopoulos

[2] SV2_Vertsioti-Zhang-Stamopoulos
